# Mapping of the chromosomal amplification 1p21-22 in bladder cancer

**DOI:** 10.1186/1756-0500-7-547

**Published:** 2014-08-18

**Authors:** Mauro Scaravilli, Paola Asero, Teuvo LJ Tammela, Tapio Visakorpi, Outi R Saramäki

**Affiliations:** Prostate Cancer Research Center, Institute of Biosciences and Medical Technology - BioMediTech, University of Tampere and Tampere University Hospital, Tampere, Finland; Department of Urology, School of Medicine, University of Tampere and Tampere University Hospital, Tampere, Finland

**Keywords:** Gene amplification, Bladder cancer, DR1, aCGH

## Abstract

**Background:**

The aim of the study was to characterize a recurrent amplification at chromosomal region 1p21-22 in bladder cancer.

**Methods:**

ArrayCGH (aCGH) was performed to identify DNA copy number variations in 7 clinical samples and 6 bladder cancer cell lines. FISH was used to map the amplicon at 1p21-22 in the cell lines. Gene expression microarrays and qRT-PCR were used to study the expression of putative target genes in the region.

**Results:**

aCGH identified an amplification at 1p21-22 in 10/13 (77%) samples. The minimal region of the amplification was mapped to a region of about 1 Mb in size, containing a total of 11 known genes. The highest amplification was found in SCaBER squamous cell carcinoma cell line. Four genes, *TMED5*, *DR1*, *RPL5* and *EVI5*, showed significant overexpression in the SCaBER cell line compared to all the other samples tested. Oncomine database analysis revealed upregulation of *DR1* in superficial and infiltrating bladder cancer samples, compared to normal bladder.

**Conclusions:**

In conclusions, we have identified and mapped chromosomal amplification at 1p21-22 in bladder cancer as well as studied the expression of the genes in the region. *DR1* was found to be significantly overexpressed in the SCaBER, which is a model of squamous cell carcinoma. However, the overexpression was found also in a published clinical sample cohort of superficial and infiltrating bladder cancers. Further studies with more clinical material are needed to investigate the role of the amplification at 1p21-22.

## Background

Bladder cancer is the fourth most common cancer in men in developed countries and the second most common malignancy of the urinary tract [1]. The majority of bladder cancer cases arise from the urothelium, the epithelium lining the inside of the bladder and these cases are thus called urothelial carcinomas. Squamous cell carcinoma of the urinary bladder is a rarer malignant neoplasm and it accounts for 3–5% of bladder cancer in Western populations [2].

Several studies have investigated the chromosomal alterations associated with development and progression of bladder cancer. Different methods to detect copy number changes, such as classical cytogenetics, interphase fluorescence *in situ* hybridization (FISH), Southern blot analysis, quantitative polymerase chain reaction (PCR)-based assays and comparative genomic hybridization (CGH) have been used [3].

Several CGH studies providing information about typical losses, gains and amplifications in bladder cancer have been published [4–8]. However, the resolution of conventional CGH is generally limited to regions greater than 10 Mb. The development of array-based technologies for CGH [9, 10] led to > 10-fold increase of the resolution and consequently to the analysis of copy number alterations at single gene level. A few array-CGH (aCGH) genome-wide studies have been performed on both clinical bladder cancers [11, 12] as well as cell lines [13]. They have highlighted copy-number alterations in smaller scale, with high accuracy of localization. Some of these genetic changes have been associated with known oncogenes or tumor suppressor genes. Loss of genetic material on chromosome 9 is one of the most frequent alteration in TCC, with 9p and 9q, often both, lost entirely or in part [14, 15]. Candidate target genes include *CDKN2A* [16], *DBCCR1* [17], and *TSC1* [18]. Deletion of 10q has been associated with *PTEN* locus [19, 20], 13q with *RB1* [21] and 17p with *TP53* [22]. Common DNA amplifications contain known or candidate oncogenes as well, including cyclin D1 (*CCND1*) at 11q13 [23, 24], *ERBB2* at 17q21 [25, 26], *E2F3* at 6p22 [27, 28], *MDM2* at 12q14 [29], and *MYC* at 8q24 [30]. Recurrent amplifications have also been found at 1q, 3p, 3q, 8p, 8q, and 12q [5, 6, 8]. Furthermore, activating mutations of oncogenes *HRAS* [31] and *FGFR3* [32] seem to be common. Gain-of-function mutations affecting *RAS* and *FGFR3* and loss-of-function mutation affecting *RB*, *PTEN* and *TP53* have also been associated with the pathological stage and/or outcome of bladder cancer [33, 34].

In this study, we report the characterization of a common amplification at chromosomal region 1p21-22. The amplicon was identified by aCGH analysis of clinical specimens obtained from bladder cancer patients and in bladder cancer cell lines.

## Methods

### Clinical samples

Freshly frozen samples from 7 bladder cancer tissues were used for this study. The samples were obtained from Tampere University Hospital and include five urothelial carcinomas, one lymphoepithelial carcinoma and one undifferentiated carcinoma. DNA was extracted using DNAzol reagent (Molecular Research Center, Inc. Cincinnati, OH), according to manufacturer’s protocol. The use of the clinical samples was approved by the ethical committee of the Tampere University Hospital.

### Cell lines

The bladder cancer cell lines UM-UC-3, TCCSUP, RT4, T24, HT-1376, J82, SCaBER, 5637, HT-1197 and SW780 were obtained from the American Type Culture Collection (ATCC, Rockville, MD, USA) and cultured according to the recommended conditions.

### Array comparative genomic hybridization

16 K cDNA microarray-slides were obtained from the Finnish Microarray DNA Centre (http://www.btk.fi/microarray-and-sequencing/) (Turku Centre for Biotechnology, University of Turku and Åbo Akademi University, Turku, Finland). The poly-L-lysine coated slides contain approximately 16000 annotated clones from sequence verified I.M.A.G.E. Consortium cDNA library in duplicate. Comparative genomic hybridization to microarray (aCGH) was done as described previously [35]. Briefly, 2 to 10 μg RsaI-digested (Fermentas UAB, Vilnius, Lithuania) DNA was labeled with Cy5-dCTP, and normal male reference DNA with Cy3-dCTP (Amersham Biosciences UK Ltd., Little Chalfont, United Kingdom), using a BioPrime Labeling Kit (Invitrogen). The sample and reference DNAs were co-hybridized overnight at +65°C, under cover slips, to microarray slides, in a final volume of 38.5 μl of hybridization mix containing 3.4 × SSC, 0.3% SDS, 1.3 × Denhardt’s (Sigma-Aldrich, St. Louis, MO), and 0.5 × DIG Blocking Buffer (Roche Diagnostics, Mannheim, Germany). After stringent washes, the slides were scanned with ScanArray4000 confocal laser scanner (Perkin Elmer, Boston, MA). Signal volumes were quantified using the QuantArray software program (Packard Bioscience, Bio- Chip Technology LCC, Billerica, MA). Data were analyzed using the cluster along chromosomes (CLAC) algorithm, as previously described and visualized using the software CGH-Miner [36].

### Fluorescence *in situ*hybridization

Human genome PAC/BAC clones were purchased from Invitrogen™ Corporation. The list of clones is shown in Table 1 and the chromosome positions are indicated according to UCSC (University of California Santa Cruz) Genome Browser, February 2009 assembly (GRCh37/h19). The clones were labeled with digoxigenin-dUTP (Roche Diagnostics) or Alexa Fluor®-dUTP (Invitrogen™) by nick translation. A pericentromeric probe for chromosome 1 labeled with FITC-dUTP was obtained from Roche. The metaphase slides from the bladder cancer cell lines were prepared using standard techniques. The slides were denatured in 70% formamide/2xSSC at 70°C for 2 min and dehydrated in an ascending ethanol series. Hybridization was performed over night at 37°C. After stringent washes, the slides were stained with antidigoxigenin-rhodamine (Roche Diagnostics) for the digoxigenin-labeled probes and embedded in an antifade solution (Vectashied, Vector Laboratories, Burlingame, CA, USA) containing 4,6-diamidino-2-phenylindole (DAPI) as counter stain. Stained slides were analyzed on an epifluorescence microscope (Olympus) and acquired images were processed using Image-Pro® image-processing software (Media Cybernetics). A total of 50 nuclei were considered for statistical analysis of the FISH signals in each experiment. An amplification was defined as a locus-specific probe/centromere ratio >2. In each experiment the hybridization efficiency of the locus-specific and centromeric probes was evaluated using 5637 bladder cancer cell line as a triploid control.

**Table 1:** **FISH mapping of 1p21-22 amplicon**

Clones	Chromosome location	Cell lines							
		**SCaBER**	**HT-1376**	**UM-UC-3**	**TCCSUP**	**RT4**	**J82**	**T24**	**5637**
**RP11-82E1**	91,116,728–91,294,152	3/4 (0.9)							3/3 (1.00)
**RP5-865 M20**	92,068,692–92,181,253	2/4 (0.54)	10/6 (1,53)		3/4 (0.86)	4/4 (0.98)	3/3 (1.00)	3/3 (1.17)	3/3 (1.00)
**RP4-621B10**	92,517,154–92,659,879	2/4 (0.54)	10/6 (1.89)		3/4 (0.86)	4/4 (1.00)	3/3 (1.00)	3/3 (1.12)	3/3 (1.00)
**RP5-1014C4**	92,854,755–93,007,879	7/4 **(2.02)**	11/6 (1.91)	4/4 (1.02)					3/3 (1.00)
**RP11-977E2**	93,042,494–93,249,510	8/4 **(2.35)**	10/6 (1.65)	3/4 (0.75)	3/4 (0.73)	4/4 (0.93)	3/3 (0.86)	4/3 (1.24)	3/3 (1.00)
**RP5-976O13**	93,529,940–93,632,330	10/4 **(3.07)**	10/6 (1.64)		3/4 (0.78)		3/3 (1.06)		3/3 (1.00)
**RP4-713B5**	93,760,493–93,865,044	11/4 **(3.02)**	10/6 (1.83)						3/3 (1.00)
**RP11-272P3**	94,980,681–95,180,686	3/4 (0.99)	11/6 (1.91)						3/3 (1.00)
**RP11-146P11**	95,983,612–96,156,674	4/4 (1.04)	10/6 (1.85)	4/4 (1.05)	3/4 (0.82)	4/4 (1.05)	3/3 (0.93)		3/3 (1.00)
**RP11-122C9**	97,095,507–97,282,884	3/4 (1.07)	10/6 (1.91)						3/3 (1.00)

The first value represents the median of signals from the locus-specific probe indicated under ‘clones’; the second value represents the median number of signal from the chromosome 1 centromeric probe. The ratio between the two values is bracketed. SCaBER cell line shows a high level amplification between the positions 92,854,755 and 93,865,044 (GRCh37/h19), whereas HT-1376 cell line shows a copy-number gain.

### RNA extraction and gene expression microarray

Total RNA from bladder cancer cell lines was collected and extracted using TRIzol reagent (Invitrogen, Carlsbad, CA, USA), according to the manufacturer’s protocol. The samples were then amplified and hybridized using the Agilent whole genome oligo microarray platform (Agilent Technologies, Palo Alto, CA, USA) and Xpress Ref ™ Human Universal Reference Total RNA (SuperArray Bioscience Corporation) was used as a reference. The resulting data files from Agilent Feature Extraction Software (version 9.5.1.1) were imported into the Agilent GeneSpring GX software (version 11.0) for further analysis. A fold-change cutoff of 2 was used to determine differential gene expression.

### Real time quantitative polymerase chain reaction (qRT-PCR)

Total RNA from bladder cancer cell lines, extracted as described above, was reverse transcribed using random hexamere primers and AMV reverse transcriptase (Thermo Scientific). Quantitative Real Time PCR was performed using Maxima SYBR Green/ROX qPCR Master Mix (Thermo Scientific) and a BioRad CFX96 ™ Real-Time PCR Detection System. Each sample was run in duplicate and expression values were normalized against TATA-binding protein (TBP). The primer sequences are shown in Table 2.

**Table 2:** **PCR primers**

Gene	Forward primer	Reverse primer
*DR1*	TGCAAGAGTGTAAAAAGTAGCATT	TGCTGCATTTGAAGCCATT
*EVI5*	AGCAGAGTGATGAGGCCAGT	CTTCACTCAGTCGGGCTTG
*RPL5*	TGGAAGAAGATGAAGATGCTTAC	GACGACATACCTCTTCTTTTTAACTTC
*TMED5*	TCACACCTTCCCTCGATAGC	AAGGTTTTGCCTTCTGGAGAG
*TBP*	GAATATAATCCCAAGCGGTTTG	ACTTCACATCACAGCTCCCC

## Results

### Identification of the common amplicon at 1p21-22

The CLAC-analysis of the aCGH data from clinical samples and bladder cancer cell lines showed a region of increased copy number at chromosome 1p21-22 in 5 of 7 total clinical samples as well as in bladder cancer cell lines, 5637, RT4, T24, SW780 and SCaBER (data not shown). According to aCGH, the common region of gain comprised of 2 Mb.

### Fine mapping of the 1p21-22 region

**Figure 1:**
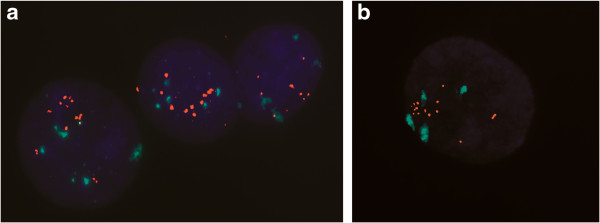
**Fluorescence** ***in situ*** **hybridization. (a)** HT-1376 cell line nuclei hybridized with the BAC clone RP11-122C9 showing copy number gain (RED: RP11-122C9, GREEN: pericentromeric chr.1), and **(b)** nuclei of SCaBER squamous cell carcinoma cell line model hybridized with the PAC clone RP4-713B5, showing a high level amplification (colors as in **a**).

The region 1p21-22 was studied in bladder cancer cell lines by FISH analysis on interphase nuclei (Figure 1). All cell lines showed increased copy number of 1p21-22 region, and SCaBER cells where the only one which showed high-level amplification of the region (Figure 1b). We extensively analyzed cell lines with the PAC/BAC clones spanning a total of 6 Mb and were able to identify a minimal region of amplification between the chromosome positions 92,940,318 and 93,828,148 (Table 2). According to UCSC Genes Feb. 2009 GRCh37/hg19, a total of 11 human genes are located within the amplicon. Nine of them are known protein-coding genes (Table 3).

**Table 3:** **Known human genes at chromosome 1 position 92,940,318 - 93,828,148 (GRCh37/h19)**

NAME	DESCRIPTION	LOCATION	GENOMIC SIZE (bp)
GFI1	Growth factor independent 1 transcription repressor (GFI1)	chr1:92,940,318 – 92,952,433	12116
EVI5	Ecotropic viral integration site 5 (EVI5)	chr1:92,974,253 – 93,257,961	283709
RPL5	Ribosomal protein L5 (RPL5)	chr1:93,297,594 – 93,307,481	9887
SNORD21	Small nucleolar RNA, C/D box 21 (SNORD21), small nucleolar RNA	chr1:93,302,846 – 93,302,940	95
SNORA66	Small nucleolar RNA, H/ACA box 66 (SNORA66), small nucleolar RNA	chr1:93,306,276 – 93,306,408	133
FAM69A	Family with sequence similarity 69, member A (FAM69A)	chr1:93,307,717 – 93,427,079	128794
MTF2	Metal response element binding transcription factor 2 (MTF2)	chr1:93,544,792 – 93,604,638	59847
TMED5	Transmembrane emp24 protein transport domain containing 5 (TMED5)	chr1:93,615,299 – 93,646,246	30948
CCDC18	Coiled-coil domain containing 18 (CCDC18)	chr1:93,646,281 – 93,744,287	98007
LOC100131564	Uncharacterized LOC100131564 (LOC100131564), non-coding RNA	chr1:93,775,666 – 93,811,368	35703
DR1	Down-regulator of transcription 1, TBP-binding (negative cofactor 2) (DR1)	chr1:93,811,478 – 93,828,148	16671

### Microarray and qRT-PCR validation

**Figure 2:**
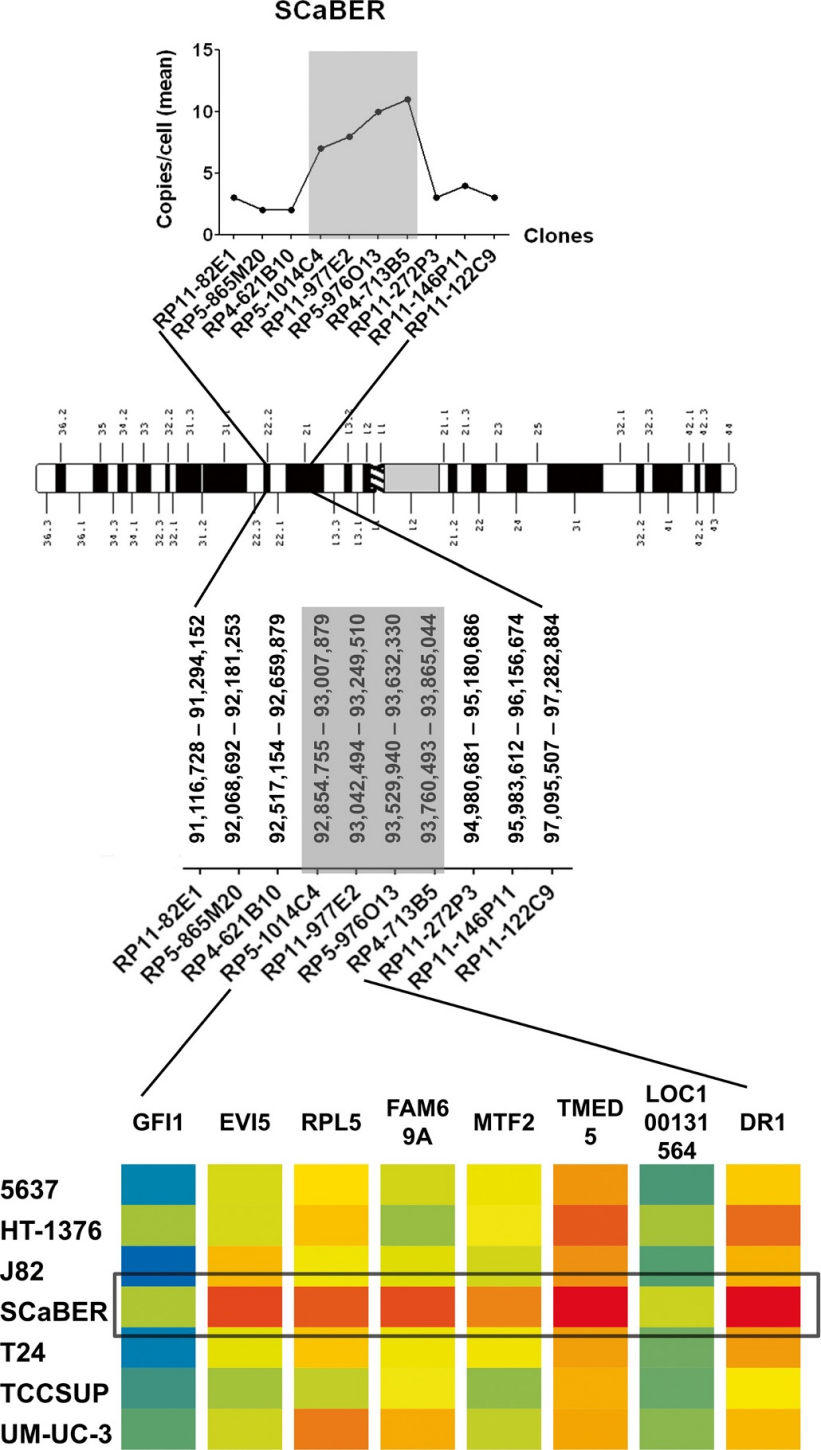
**Fine mapping the region of amplification.** Chromosome 1 ideogram showing the region of amplification according to aCGH (above), the FISH scoring data on SCaBER cell lines indicating the minimal region of amplicon (in gray), and (below) an expression heatmap of the genes at chromosome 1, position 92,940,318 – 93,828,148 (red: overexpression, blue: underexpression), showing significant relative overexpression of *TMED5*, *DR1*, *EVI5* and *RPL5* in the SCaBER cell line.

**Figure 3:**
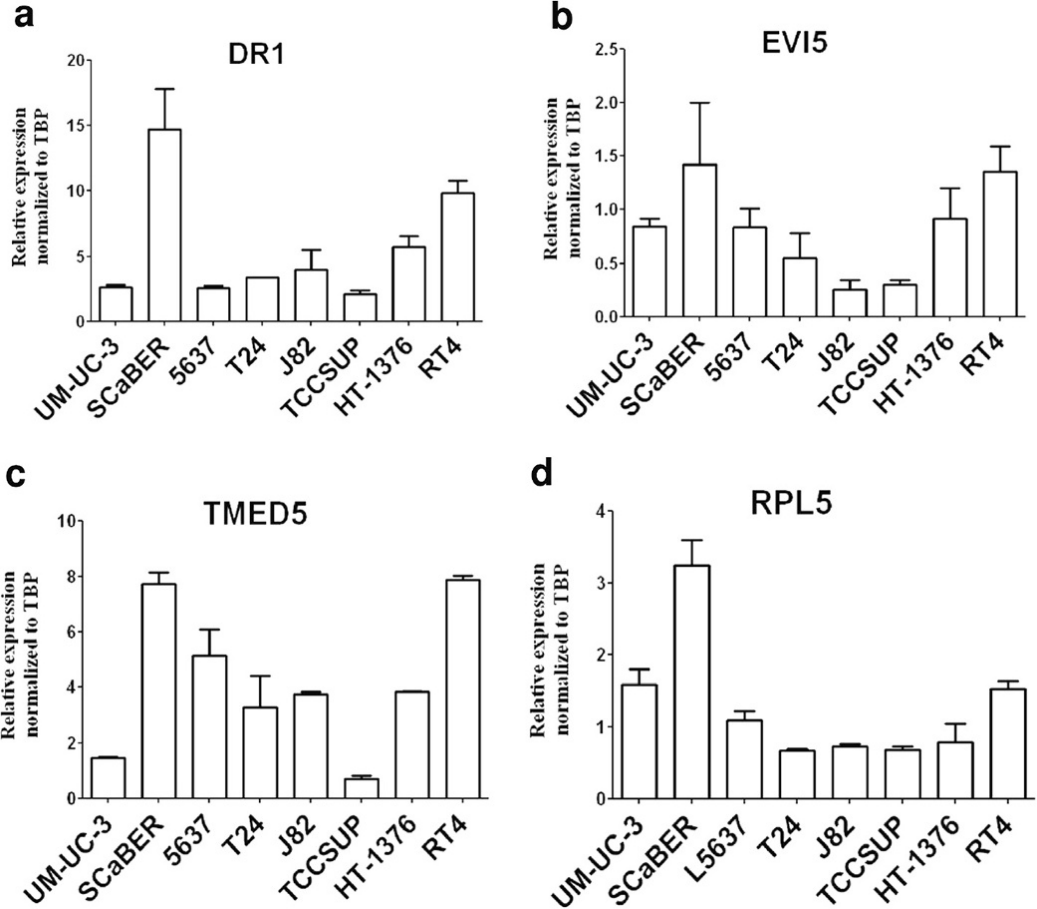
**qRT-PCR validation of microarray expression data.** *DR1* **(a)**, *EVI5* **(b)**, *RPL5* **(c)** and *TMED5* **(d)**, showing the highest level of expression in the SCaBER model, when compared to the other cell lines tested. The expression values of the genes were normalized against *TBP*.

**Figure 4:**
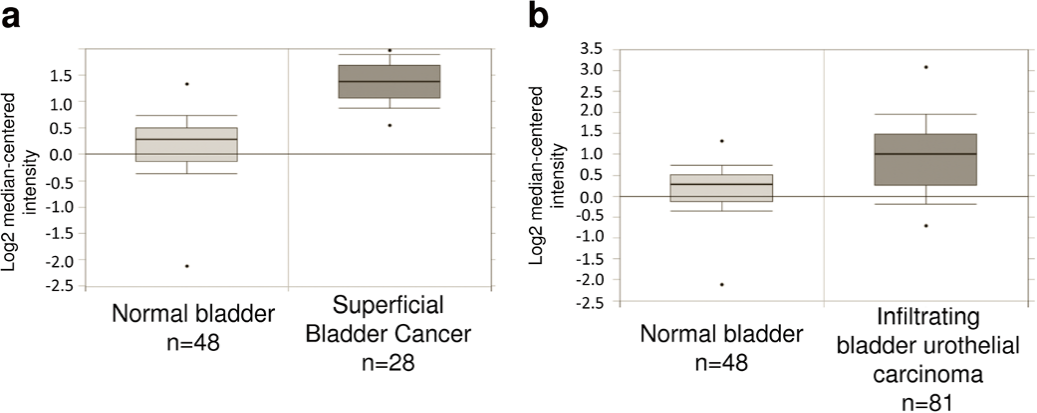
**DR1 expression in bladder cancer according to Oncomine.** Statistically significant (p < 0.0001) upregulation of *DR1* expression was found in superficial **(a)** and infiltrating **(b)** bladder cancer, when compared to normal bladder. A total of 157 samples were used in the Sanchez-Carbayo study (Sanchez-Carbayo et al., 2006).

The analysis of gene expression by microarray showed significant overexpression of 4 genes, namely *DR1*, *EVI5*, *RPL5* and *TMED5* only in the SCaBER, which harbors the highest level of amplification of the region (Figure 2). The results were validated by qRT-PCR and confirmed the overexpression of the genes in SCaBER, as compared to all the other cell lines (Figure 3). In addition, Oncomine database analysis for *DR1* expression in bladder cancer revealed a statistically significant (P < 0.0001) upregulation of the gene in clinical samples of both superficial and infiltrating bladder cancer, when compared to normal bladder [37] (Figure 4). *TMED5* showed significant upregulation in superficial bladder cancer, when compared to normal, whereas *RPL5* and *EVI5* did not show significant changes of expression levels in the same dataset.

## Discussion

In this study, aCGH technology was utilized to identify new regions of amplifications in bladder cancer. Recurrent amplification was found in chromosomal locus 1p21-22. Subsequently, the locus was fine-mapped and characterized in the bladder cancer cell lines. Of the cell lines SCaBER showed the highest amplification of the region, thus it was used for mapping the amplicon. Fine mapping with the SCaBER model, the region was defined to ~1 Mb of size, containing 11 genes.

cDNA microarray and qRT-PCR analyses were used to measure the expression of these genes in bladder cancer cell lines. *DR1*, *EVI5*, *RPL5*, and *TMED5* showed overexpression in SCaBER compared to the other cell lines. *DR1* was found to be the most significantly overexpressed of the examined genes. Since SCaBER is a squamous cell carcinoma cell line, we wished to interrogate whether *DR1* is overexpressed also in the urothelial carcinoma. We utilized Oncomine database of clinical samples, which showed overexpression of *DR1* also in superficial and infiltrating bladder cancer.

*DR1* is also known as *NC2beta* and has been shown to bind *DRAP1* to repress RNA polymerase II gene transcription [38]. Despite targeting the general transcription machinery, only a subset of mRNAs has been shown to respond to the *DR1/DRAP1* inhibition [39] and the opposite transcription inducing effect of DR1/DRAP1 has also been shown for some mRNAs, suggesting the possibility of a specific regulatory effect [40].

According to Oncomine database *DR1*, *EVI5*, *TMED5* and *RPL5* are co-amplified also in brain [41–43], colon [44], lung cancer [45] and melanoma [46], indicating that amplification of 1p21-22 may be a recurrent alteration in several different types of cancers.

## Conclusions

We have identified and mapped a common chromosomal amplification at 1p21-22 in bladder cancer. Squamous cell carcinoma cell line SCaBER, which had the highest level of amplification of the region, showed overexpression of *DR1*. In a published data set, *DR1* was also overexpressed in clinical samples of superficial and infiltrating bladder cancers, suggesting that *DR1* is a putative target for the amplification. Further studies are needed to assess the role of the amplification at 1p21-22 in bladder cancer.
